# Supplementary material for: Collagen piezoelectricity in osteogenesis imperfecta and its role in intrafibrillar mineralization
Source: Commun Biol. 2022 Nov 11;5:1229. doi: 10.1038/s42003-022-04204-z (PMC9652255; doi:10.1038/s42003-022-04204-z)
Supplement: Supplementary file 2 — Supplementary Information [file 42003_2022_4204_MOESM2_ESM.pdf]

Supplementary Information

Collagen Piezoelectricity in Osteogenesis Imperfecta and Its Role in Intrafibrillar Mineralization

Jinha Kwon, and Hanna Cho*

Jinha Kwon, Prof. Dr. Hanna Cho
Mechanical and Aerospace Engineering, The Ohio State University, 201 W 19th Ave, Columbus, OH 43210, USA
*E-mail: cho.867@osu.edu

Quantitative PFM data at the gap and overlap regions

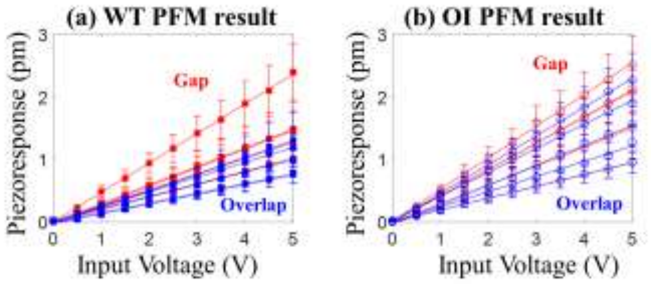

Supplementary Fig. 1. The PFM result of point measurements at the gap (red) and overlap (blue) regions of five (a) WT and (b) OI collagen fibrils, respectively.

Supplementary Fig. 1 shows piezoresponse of five WT and OI collagen fibrils with respect to the input AC voltage from 0V to 5V in 0.5V steps. In each step, piezoresponse measurement was repeated ten times on gap (red) and overlap (blue) regions. In the WT collagen, the piezoresponses on the gap region are around two times higher than the piezoresponses on the overlap region, while OI collagen fibrils do not exhibit a noticeable difference between the two regions. The average piezoresponse of WT and OI collagen fibrils with their coefficients are displayed in Fig. 2 in the main manuscript.

Osteogenesis imperfecta (OI)

Osteogenesis imperfecta (OI) is a genetic bone disorder, approximately 6 to 7 per 100,000 people worldwide suffer from, associated with a mutation in type I collagen $\alpha 1$ (COL1A1) and $\alpha 2$ (COL1A2) chains[1]. The modification of collagen molecules in OI causes structural changes and abnormal mineralization, which results in a decrease of ultimate strength and fracture toughness. This reveals bone growth deficiency and high fragility at the whole bone level[2]. Even though more than 800 COL1A1 and COL1A2 mutations have been reported in OI, they can be classified by 27 types with an overall spectrum of disease severity ranging from a perinatal lethal to mild disease[3]. In this study, G610C knock-in mouse was investigated, which alters the gly-610 codon (GGT) to a cysteine (TGT) codon for COL1A2. This model, known as Amish OI, represents a human OI type IV phenotype observed from the large population of

the Old Order Amish kindred, Lancaster County, Pennsylvania[4]. Even though *BrtlIV* mouse and osteogenesis imperfecta murine (*oim*) also have been widely used to study OI type IV phenotype[5–7], those models may not be appropriate to represent the human case. For instance, the *BrtlIV* mouse has triple-helical glycine substitutions in the COL1A1 chain, and an OI phenotype pattern of the mutation in the COL1A1 appears to be different from the pattern in the COL1A2. Also, the COL1A1 mutation is more severe than the mutation in the COL1A2[4]. On the other hand, *oim* represents an OI type IV phenotype with COL1A2 mutation. However, the *oim* mouse exhibits an atypical phenotype which attributed to its homotrimers consisting of three α1 chains, rarely observed in a human case[4,7]. In this regard, the Amish OI model represents a unique translational model for human cases, and it has been studied to evaluate phenotype variability and investigate potential therapeutic strategies.

Resonance-enhanced PFM

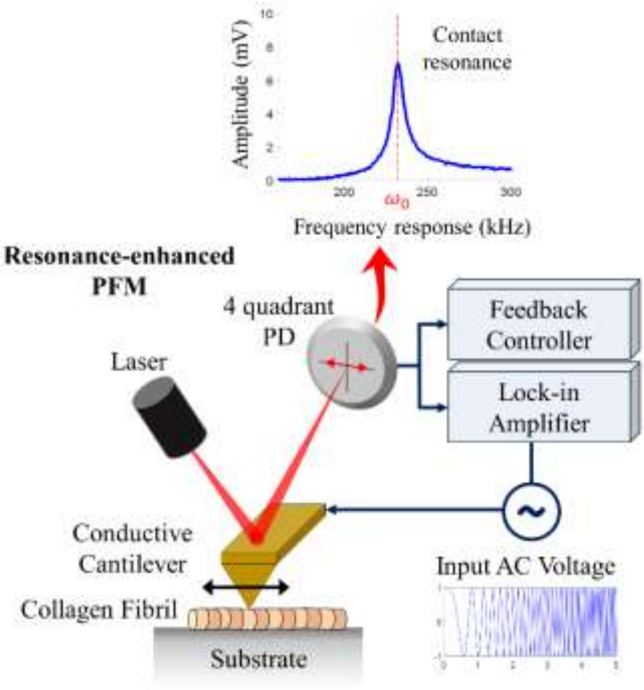

Supplementary Fig. 2. A schematic of the resonance-enhanced PFM. The piezoresponse of a collagen fibril is amplified at the contact resonance frequency, which can be found by frequency sweep from low to high frequency of an input AC voltage.

The resonance-enhanced PFM technique utilizes contact resonance to amplify the piezoelectric signal[8]. Supplementary Fig. 2 illustrates a schematic of the resonance-enhanced PFM. The cantilever tip keeps in contact with a specimen during scanning, and AC voltage is applied through a conductive cantilever tip. In this study, the resonance-enhanced PFM in the lateral direction (in-plane), using the first lateral vibrational mode of cantilever, was conducted to obtain a shear piezoelectric coefficient of collagen. The frequency response curve was obtained for every single PFM measurement to find the contact resonance frequency and a set of representative data is shown in Figs. 7b-c in the main manuscript.

Dual AC Resonance Tracking piezo force microscopy (DART-PFM)

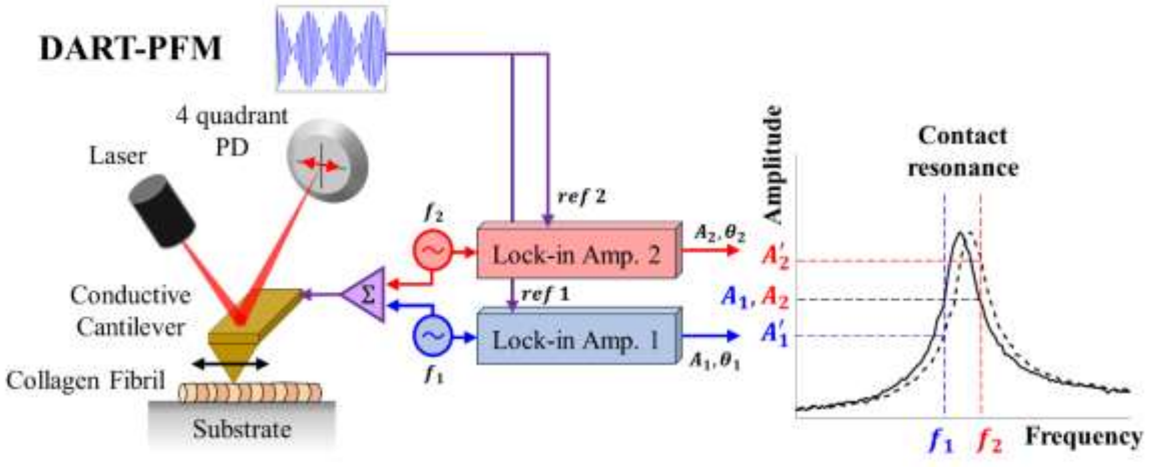

Supplementary Fig. 3. Dual AC Resonance Tracking piezo force microscopy (DART-PFM) schematic: DART-PFM is a technique designed to mitigate a crosstalk effect between topography and piezoresponse by utilizing two frequencies adjacent to the contact resonant frequency. When the contact resonant frequency shifts upward due to a topographical change, A_1 moves down to A_1', and A_2 moves up to A_2'. The feedback controller calculates the change in the $A_2 - A_1$ signal and adjusts the operating frequencies to maintian the $A_2 - A_1$ signal to be zero. As a result, the change of the contact resonant frequency can be tracked, and the crosstalk effect can be compensated.

Dual AC Resonance Tracking PFM (DART-PFM) is a technique designed to mitigate the crosstalk effect by which the topographical change during PFM scanning influences the final PFM result[9]. Since the resonance-enhanced PFM operates at the contact resonant frequency, a change of this reference frequency can cause an artifact in the PFM result. To compensate the crosstalk effect, DART-PFM utilizes two separate frequencies with two lock-in amplifiers as

illustrated in supplementary Fig. 3. Those two frequencies are set near the contact resonant frequency, slightly lower and higher than the resonant frequency, and the cantilever deflection signals at these two frequencies are delivered to two lock-in amplifiers. If the contact resonant frequency is changed during the PFM scanning due to the crosstalk effect, this change can be compensated by tracking the change of the two amplitudes. For instance, when the contact resonant frequency moves upward as indicated in the dashed line in supplementary Fig. 3, A_1 moves down to A_1' and A_2 moves up to A_2'. The system calculates the change in the $(A_2 - A_1)$ signal and shifts the resonant frequency to make the $(A_2 - A_1)$ zero by a feedback controller. As a result, the change of the contact resonant frequency can be tracked and the crosstalk effect is compensated.

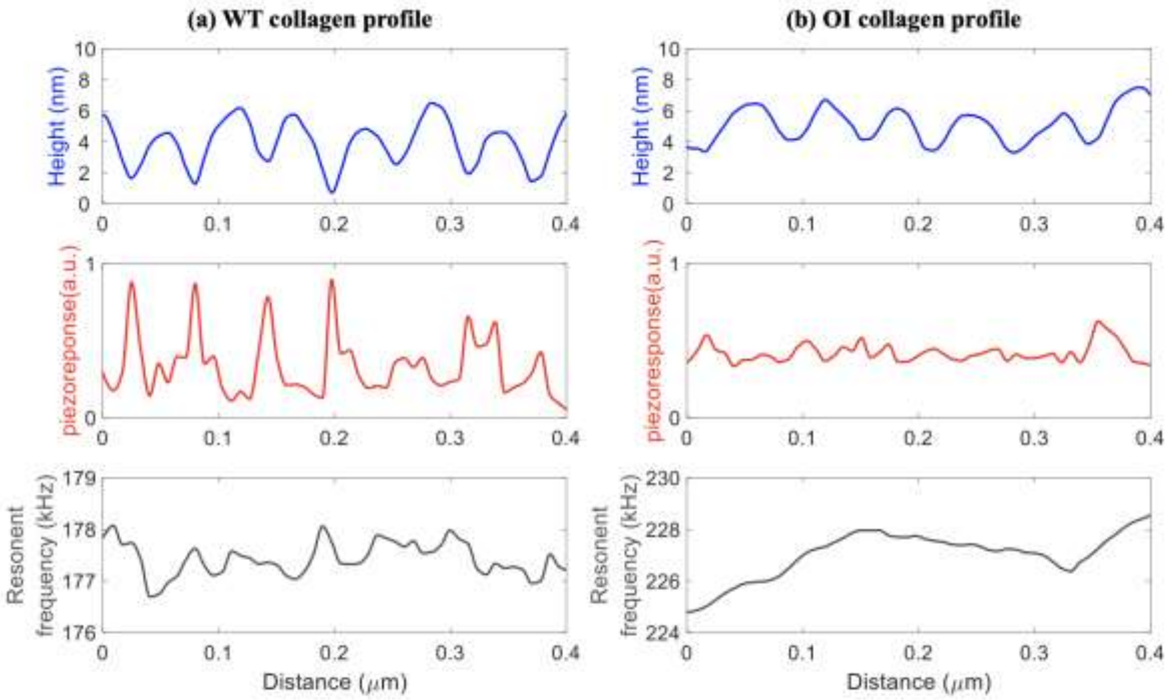

Supplementary Fig. 4. Height, piezoresponse, and resonant frequency profile of (a) WT collagen and (b) OI collagen obtained from DART-PFM.

In this study, we used DART-PFM to obtain the piezoresponse profile of a collagen fibril in the WT and OI model with the minimzed crosstalk effect. To confirm the effect of frequency shift, supplementary Fig. 4 shows the height, piezoresponse, and resonant frequency profiles along

with the WT and OI collagen fibril. Although, in both collagens, the resonant frequency was shifted due to the changes in the topography and mechanical properties along the fibrils, DART-PFM was able to track and compensate for the resonant frequency shifting. Also, the resonant frequency shifting patterns do not match the height and piezoresponse profiles, indicating that the piezoresponse of each collagen does not stem from the resonant frequency change. Moreover, the piezoresponse patterns in the profile of both collagen samples acquired by the DART-PFM (Fig. 2c and f) are well supported by the quantitative results obtained by the quantitative point measurements of PFM (Fig. 3).

Error propagation in quantitative PFM

VInvOLS was measured 30 times to be 172.94 $\pm$ 25.53 nm/V. In addition, the cantilever specification (3XC-GG, OPUS®) described that the length of cantilever (L) is 500 μm and the height (h) is 14 μm. Here, we assumed that the height ranges from 12 to 16 μm and length ranges from 490 to 410 um, indicating 95% of confidential interval. Therefore, the standard deviation of the height (δh) and length (δL) were estimated to be 1.02 and 5.10 μm, respectively. If the collagen fibril is not aligned perpendicularly to the cantilever coordinates, it would cause an orientation error. Thus, it was assumed that a collagen fibril would be misaligned by 10° $\pm$10°. Additionally, the PFM signal (A_{mv}) was measured ten times at each setup to obtain $\overline{A_{mv}}$ and δA_{mv} values. As a result, the average of calibrated PFM amplitude ($\overline{A_{pm}}$) can be determined by

$$\overline{A_{pm}} = \frac{\overline{A_{mv}}}{Q} \cdot \frac{\overline{VinvOLS} \cdot 3\bar{h}}{2\bar{L}} \cdot cos\bar{\theta} \qquad \text{Eqn. S1}$$

The standard deviation of the calibrated PFM amplitude can be obtained by

$$\delta A_{pm} = |\overline{A_{pm}}| \cdot \sqrt{\left(\frac{\delta A_{mv}}{\overline{A_{mv}}}\right)^2 + \left(\frac{\delta VinvOLS}{\overline{VinvOLS}}\right)^2 + \left(\frac{\delta h}{\bar{h}}\right)^2 + \left(\frac{\delta L}{\bar{L}}\right)^2 + \left(\frac{\delta\theta \cdot sin\theta}{cos\,\bar{\theta}}\right)^2} \qquad \text{Eqn. S2}$$

Consequently, the error of each term was carefully considered in this work, and the errors of quantitative PFM result were estimated in a range of 20-35%.

Piezoelectric tensor for a collagen fibril

The piezoelectric tensor of a collagen fibril is described by Eqn. S6[12].

$$d_{ij} = \begin{bmatrix} 0 & 0 & 0 & d_{14} & d_{15} & 0 \\ 0 & 0 & 0 & d_{15} & -d_{14} & 0 \\ d_{31} & d_{31} & d_{33} & 0 & 0 & 0 \end{bmatrix} \qquad \text{Eqn. S3}$$

In this work, The AFM cantilever was aligned with the y-axis to be perpendicular to the collagen fibrillar direction (z-axis). In this measurement setting, only d_{15} piezoelectric coefficient can be assessed by the lateral PFM (in-plane), while other piezoelectric coefficients (d_{31}, d_{33}, and d_{14}) cannot be obtained because the electric potential cannot be applied along the fibril's longitudinal direction to measure d_{31} and d_{33} and a collagen fibril was fixed on the substrate resulting in nullification in the d_{14} direction.

PFM experiment result of PPLN sample for validation of the calibration

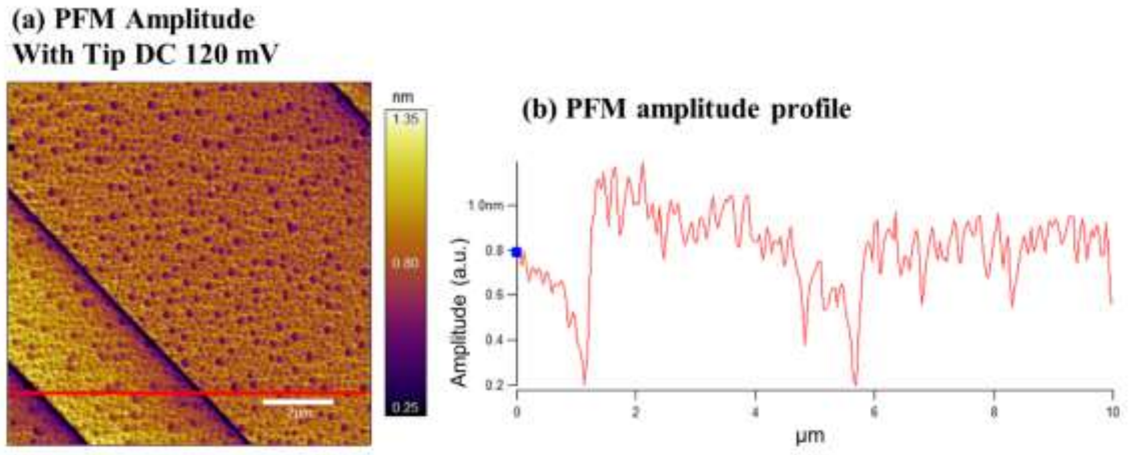

Supplementary Fig. 5. (a) PFM amplitude map of a PPLN sample scanned by vertical resonance-enhanced PFM. (out-of-plane) (b) PFM amplitude profile along with the red line in (a). In these results, VinvOLS (185.6 nm/V) was applied to convert the photodetector voltage to the cantilever tip movement.

In order to validate our calibration method, we conducted the resonance-enhanced PFM on a PPLN (Periodically Poled Lithium Niobate) sample that has typically 10~20 pm/V (d_{33}) piezoelectric coefficient as provided by Asylum Research[13]. Here, we used the same type of cantilever that used for the collagen sample (3XC-GG, OPUS®). The static sensitivity of the cantilever VinvOLS was obtained as 185.6 nm/V from the force curve on a sapphire surface. In addition, the cantilever stiffness and Q factor in free vibration were 2.1 N/m and 70.1, respectively, which were obtained from the thermal spectrum of the cantilever. On the PPLN surface, we obtained contact resonant frequency, Q factor, and contact stiffness as 76.87 kHz, 67.6, and 24.25 N/m, respectively. By numerically solving the beam vibration equation[14], we obtained the ratio between the dynamic and static sensitivities of the cantilever as shown in Eqn.4 as $\lambda = 0.96$. Therefore, we assumed that the dynamic sensitivity equals the static sensitivity of the cantilever in this PFM setting. Because vertical PFM is greatly affected by the electrostatic force, 120 mV DC voltage (V_{dc}) in addition to 1 V AC input voltage (V_{ac}) was applied to compensate the surface potential (V_{sp}) to eliminate the electrostatic contribution based on Eqn. 2. Supplementary Fig. 5a displays the PFM amplitude map of the PPLN, and Supplementary Fig. 5b demonstrates the PFM amplitude profile along with the redline in Supplementary Fig. 5a. By calibrating the measured PFM amplitude, obtained piezoelectricity in d_{33} of the PPLN was obtained to be around 12 pm/V, which is in the range of the specification, supporting the validation of our calibration method.

Supplementary References

1. Carriero, A. *et al.* How Tough Is Brittle Bone? Investigating Osteogenesis Imperfecta in Mouse Bone. *J. Bone Miner. Res.* **29**, 1392–1401 (2014).
2. Vanleene, M. *et al.* Ultra-structural defects cause low bone matrix stiffness despite high mineralization in osteogenesis imperfecta mice. *Bone* **50**, 1317–1323 (2012).
3. Fan, Z., Smith, P. A., Harris, G. F., Rauch, F. & Bajorunaite, R. Comparison of Nanoindentation Measurements Between Osteogenesis Imperfecta Type III and Type IV and Between Different Anatomic Locations (Femur/Tibia versus Iliac Crest). *Connect. Tissue Res.* **48**, 70–75 (2007).
4. Daley, E. *et al.* Variable bone fragility associated with an Amish COL1A2 variant and a knock-in mouse model. *J. Bone Miner. Res.* **25**, 247–261 (2010).
5. Kemp, A. D., Harding, C. C., Cabral, W. A., Marini, J. C. & Wallace, J. M. Effects of tissue hydration on nanoscale structural morphology and mechanics of individual Type I collagen fibrils in the Brtl mouse model of Osteogenesis Imperfecta. *J. Struct. Biol.* **180**, 428–438 (2012).
6. Wallace, J. M., Orr, B. G., Marini, J. C. & Holl, M. M. B. Nanoscale morphology of Type I collagen is altered in the Brtl mouse model of Osteogenesis Imperfecta. *J. Struct. Biol.* **173**, 146–152 (2011).
7. Andriotis, O. G. *et al.* Structure–mechanics relationships of collagen fibrils in the osteogenesis imperfecta mouse model. *J. R. Soc. Interface* **12**, 20150701 (2015).
8. Jesse, S., Mirman, B. & Kalinin, S. V. Resonance enhancement in piezoresponse force microscopy: Mapping electromechanical activity, contact stiffness, and Q factor. *Appl. Phys. Lett.* **89**, 022906 (2006).
9. Rodriguez, B. J., Callahan, C., Kalinin, S. V. & Proksch, R. Dual-frequency resonance-tracking atomic force microscopy. *Nanotechnology* **18**, 475504 (2007).

10. D'Costa, N. P. & Hoh, J. H. Calibration of optical lever sensitivity for atomic force microscopy. *Rev. Sci. Instrum.* **66**, 5096–5097 (1995).

11. Peter, F., Rudiger, A., Szot, K., Waser, R. & Reichenberg, B. Sample-tip interaction of piezoresponse force microscopy in ferroelectric nanostructures. *IEEE Trans. Ultrason. Ferroelectr. Freq. Control.* **53**, 2253–2260 (2006).

12. de Jong, M., Chen, W., Geerlings, H., Asta, M. & Persson, K. A. A database to enable discovery and design of piezoelectric materials. *Sci. Data.* **2**, 150053 (2015).

13. https://afm.oxinst.com/assets/uploads/products/asylum/documents/Piezoresponse-Force-Microscopy-AFM-web.pdf.

14. Balke, N. *et al.* Quantification of surface displacements and electromechanical phenomena via dynamic atomic force microscopy. *Nanotechnology* **27**, 425707 (2016).
