# Supplementary material for: Collagen piezoelectricity in osteogenesis imperfecta and its role in intrafibrillar mineralization
Source: Commun Biol. 2022 Nov 11;5:1229. doi: 10.1038/s42003-022-04204-z (PMC9652255; doi:10.1038/s42003-022-04204-z)
Supplement: Supplementary file 4 — Supplementary Data [file 42003_2022_4204_MOESM4_ESM.zip › Supplementary Data files/README.docx]

This is the description of additional supplementary files.

- **Raw data**
  - WT LPFM_MI.xlsx 🡪 Lateral PFM raw data of mineralized wild type bone (MI)
  - WT_PFM.xlsx 🡪 Lateral PFM raw data of demineralized wild type collagens (WT)
  - OI_PFM.xlsx 🡪 Lateral PFM raw data of demineralized osteogenesis imperfecta collagens (OI)
  - WTOIPFM_Data.mat 🡪 Dataset of calibrated WT, OI, MI PFM data for MATLAB. Detailed description for each column of the dataset in WTMIPFM_New.m
  - WTOI_Each_Data.mat 🡪 Lateral PFM raw data of WT, OI for MATLAB. Detailed description for each column of the dataset in WTMIPFM_New.m
  - MI_Each_Data.mat 🡪 Lateral PFM raw data of MI for MATLAB.
- **Figure 2**
  - WTPFM_Profile.m 🡪 MATLAB code to draw Figure 2c
  - WTPFM_Profile.mat 🡪 MATLAB data to draw Figure 2c
  - OIPFM_Profile.m 🡪 MATLAB code to draw Figure 2f
  - OIPFM_Profile.mat 🡪 MATLAB data to draw Figure 2f
- **Figure 3, Supplementary Figure 1**
  - Calibrated_WTOIPFM_final.m 🡪 MATLAB code to draw Figure 3a, 3b
  - Calibrated_WTOIPFM.mat 🡪 MATLAB data to draw Figure 3a, 3b
  - WTOIPFM_Bar_JMP_basedon_bone.m 🡪 MATLAB code to draw Figure 3c
- **Figure 6**
  - WTOI_Width.m 🡪 MATLAB code to draw Figure 6a
  - WTOI_Width.mat 🡪 MATLAB data to draw Figure 6a
  - WTMIPFM_New.m 🡪 MATLAB code to draw Figure 6f
- **Figure 7**
  - Resonant_curvefitting_Qfactor.m 🡪 MATLAB code to draw Figure 7b, 7c
  - Resonant_curvefitting_Qfactor_Data.mat 🡪 MATLAB data to draw Figure 7b, 7c
  - Q_factor_calculator.m 🡪 MATLAB code to calculate Q factor from PFM data
- **Supplementary Figure 4**
  - WT_DART_Freqshift_subplot.m 🡪 MATLAB code to draw Supplementary Figure 4a
  - WT_DART_Freqshift.mat 🡪 MATLAB data to draw Supplementary Figure 4a
  - OI_DART_Freqshift_subplot.m 🡪 MATLAB code to draw Supplementary Figure 4b
  - OI_DART_Freqshift.mat 🡪 MATLAB data to draw Supplementary Figure 4b
